# Advantages and problems of nonlinear methods applied to analyze physiological time signals: human balance control as an example

**DOI:** 10.1038/s41598-017-02665-5

**Published:** 2017-05-26

**Authors:** Wolfram Müller, Alexander Jung, Helmut Ahammer

**Affiliations:** 1 Institute of Biophysics, Center for Physiological Medicine, Medical University of Graz, Harrachgasse 21/IV, 8010 Graz, Austria; 2 Institute of Bioengineering, Aachen University of Applied Sciences, Heinrich-Mußmann-Straße 1, 52428 Jülich, Germany

## Abstract

Physiological processes are regulated by nonlinear dynamical systems. Various nonlinear measures have frequently been used for characterizing the complexity of fractal time signals to detect system features that cannot be derived from linear analyses. We analysed human balance dynamics ranging from simple standing to balancing on one foot with closed eyes to study the inherent methodological problems when applying fractal dimension analysis to *real-world* signals. Higuchi dimension was used as an example. Choice of measurement and analysis parameters has a distinct influence on the computed dimension. Noise increases the fractional dimension which may be misinterpreted as a higher complexity of the signal. Publications without specifying the parameter setting, or without analysing the noise-sensitivity are not comparable to findings of others and therefore of limited scientific value.

## Introduction

Human physiology is predominated by nonlinear dynamical systems resulting in fractal patterns^1–5^. Fractals show self-similarity over a range of scales^6, 7^. For studying fractal physiological time signals, nonlinear measures including fractal dimensions, entropies, and Hurst’s exponents are widely used^1, 8^. They characterize the complexity of the signal’s morphology, independently of the absolute signal elongations and enable direct investigations without phase space reconstruction.

Every day, each of us solves thousands of postural and balance control tasks of various levels of difficulty with seemingly little effort in amazingly elegant way. Because of the large number of degrees of freedom of the body’s mechanics, the nonlinear coupling of the body segments, and the complexity of neuro-muscular control mechanisms, balance control necessitates extremely sophisticated senso-motoric processes, even for every-day movements. We synergistically use our visual, vestibular, and somatosensory systems for the feedback loops necessary to perform these complex motion tasks, to coordinate and optimise the interactions of motions of body parts, to perform two or more motion tasks in parallel such as walking and balancing a tray with a heavy load of dishes on it. The human postural control system has important basic functions that are necessary for our various interactions with the external world: it builds up posture against gravity, it maintains balance, it controls the orientation and position of body segments with respect to the internal capabilities and to the boundary conditions given by the external world, and it creates a frame for our perception of the world surrounding us^9^.

To stay in balance can be improved substantially by training. This holds true for all persons including athletes and artists on the high performance end, and for older persons and patients who suffer from balance control deficits, too^10^. The comprehensive balance test series (seven balance tasks lasting one minute each) we are presenting here is capable of quantifying balance capabilities ranging from simple standing on both feet to balancing on one ball of the foot with open eyes (except for some acrobats, the latter task performed with closed eyes cannot be solved anymore - because it is too difficult).

Besides traditional linear analysis methods, nonlinear methods are frequently used for studying a wide range of physiological and pathophysiological processes including disorders related to aging. Among them are: postural control and gait^11–18^, heart rate variability^19–22^, brain activity^22–25^, and breathing^26^. Unfortunately, in many studies measurement and analysis parameters, and signal-to-noise ratios (SNR), that have decisive influence on the outcome, are not reported sufficiently.

The signal’s absolute values of elongations are not of relevance for these nonlinear measures; in other words, multiplying the time signal with a constant factor does not change the values of these measures. Even very small signal elongations may influence the result substantially. Therefore, it is to be expected that noise, which accompanies any measurement, may have a pronounced influence on the results^16, 27^. When the physiological control process is very regular, for example when the pace of the heart shows little variation, the nonlinear measures for the signal complexity may mirror the noise rather than the actual control process. This also holds true for postural and balance control signals of the centre of pressure sway measured on a force plate. The set-up for such measurements can be modified easily to study various cases of complexity, noise influences, and effects of measurement and analysis parameter settings.

As an example of nonlinear analysis, the Higuchi dimension *D*_*H*_^28^ was used to compare the postural sway complexity of a series of balance tasks which we have developed to analyse balance abilities ranging from simple standing on both feet with eyes open to the difficult task of balancing on one foot with closed eyes. This study design is used here to analyse the important influences of noise and of the parameter settings on the computed fractal dimension. We used the Higuchi dimension for this exemplary analysis because this nonlinear measure is widely used and usually shows a high linearity in the double-logarithmic plot even for limited lengths of data sets.

## Results

**Figure 1:**
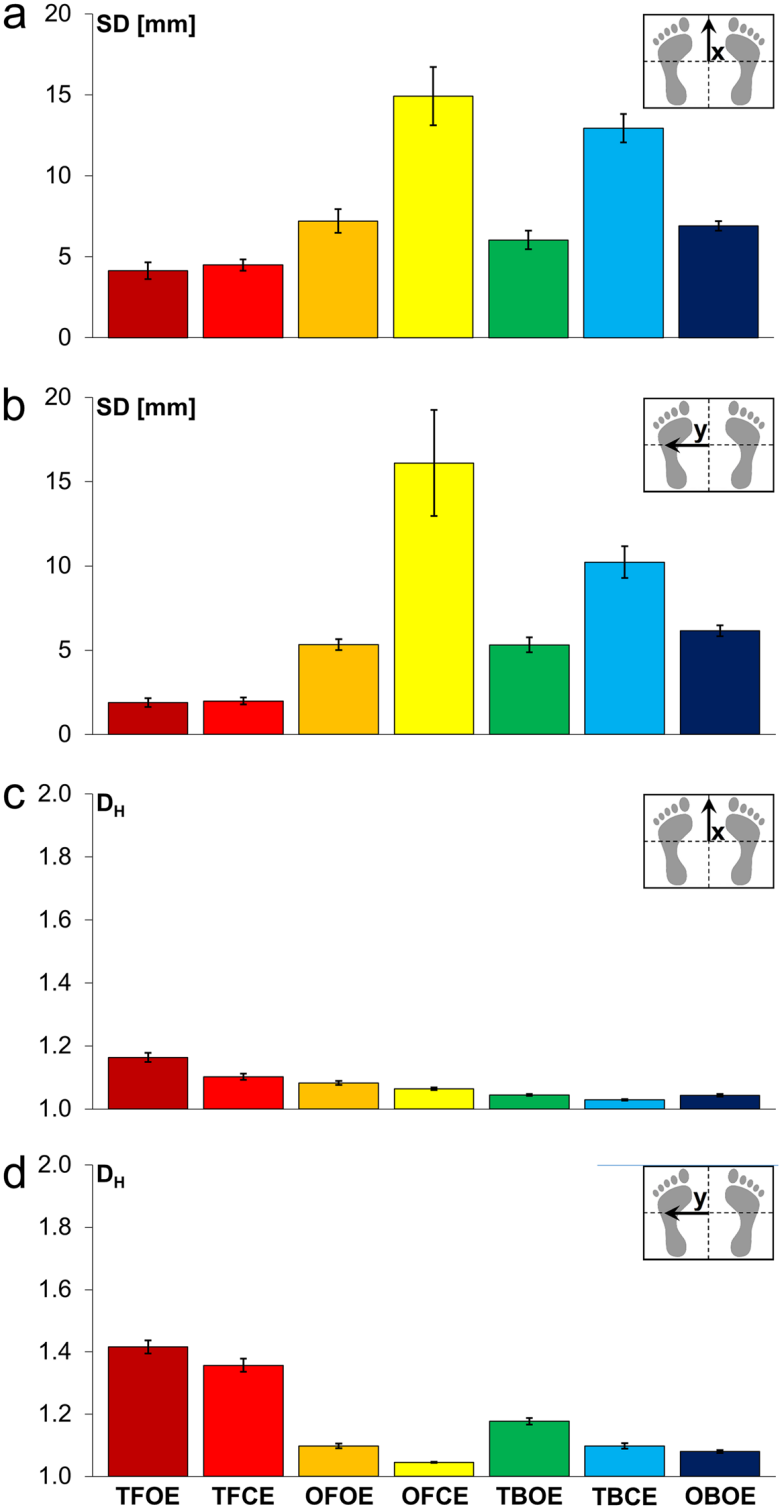
Linear and nonlinear analyses of the seven balance tasks. Abbreviations: TF (two feet), OF (one foot), TB (two balls), OB (one ball), OE (open eyes), CE (closed eyes). Significant differences (Kruskal-Wallis test) are presented in Tables 1 and 2. The columns represent the mean values of the fifteen test persons who participated in the test series. Standard errors of mean (SEM) are also indicated. Analyses of the center of pressure (COP) sway in terms of standard deviations (SD) are shown in (**a**,**b**). (**a**) represents the SDs of the sway of the COP in (anterior-posterior) *x*-direction, and (**b**) in (medio-lateral) *y*-direction. The columns in (**c**) represent the Higuchi dimensions *D*_*H*_ with respect to the x-component of the COP sway and (**d**) shows the *D*_*H*_ values according to the *y*-component.

The results of the centre of pressure (COP) sway time signal analysis with respect to x-direction (anterior-posterior) and y-direction (medio-lateral) of the ground reaction force plate are shown in Fig. 1. The seven tasks lasted 60 s each and included standing on two feet (TF), two balls (TB), one foot (OF), and on one ball (OB). The tasks were performed with open eyes (OE) and, except for the latter (which turned out to be too difficult), also with closed eyes (CE). Columns in Fig. 1a represent the means (of one attempt of each of the 15 test persons) of the standard deviations (SD) of the COP elongations in *x*-direction, and Fig. 1b represents those obtained in *y*-direction. Figsure 1c,d represent the respective means of the Higuchi dimensions of the COP sway time signals. The small standard errors of the means (SEM) are also indicated. Ranking of the balance tasks according to increasing means of SDs (n = 15) was not in line with the ranking according to decreasing *D*_*H*_. Largest mean of SD was found for OFCE in both directions. *D*_*H*_ in *y*-direction was lowest for OFCE. The lowest *D*_*H*_ in *x*-direction was found for standing on two balls with closed eyes (TBCE). Table 1 shows significant differences between tasks according to the SDs of the elongations and Table 2 according to *D*_*H*_. COP sway time signals in *x*-direction resulted in 14 significant differences (out of 21 comparisons between all tasks) when SD was used for the analysis, and 15 were found when *D*_*H*_ was used. In *y*-direction, 15 and 16 comparisons resulted in significant differences, respectively. Significances of task comparisons based on SD-analysis and on *D*_*H*_-analysis did not correspond (indicated with brackets) in nine cases for *x*-direction COP sway, and in five cases for *y*-direction COP sway. All 15 participants performed each task three times. The first successful attempt was used for the analysis shown here. Three time signals out of 15 × 7 = 105 (two times OBOE and one time OFCE) lasted less than 60 s, but longer than the minimum criterion of 30 s (the longest time signal lasting between 30 s and 60 s was taken). When considering all 315 attempts, 19 did not last 60 s (11 such cases for OBOE, five cases for OFCE, two cases for TBCE, and one case for OFOE).

**Table 1:** Significant differences between balance task results for standard deviations SD of balance motion elongations.

	TFOE	TFCE	OFOE	OFCE	TBOE	TBCE	OBOE
**TFOE**		n	**	**	(n)	**	**
**TFCE**	n		(*)	**	(n)	**	**
**OFOE**	**	**		(**)	(n)	**	(n)
**OFCE**	**	**	**		(**)	(n)	**
**TBOE**	**	(**)	(n)	**		**	n
**TBCE**	**	**	(**)	(n)	**		(**)
**OBOE**	**	**	n	**	(n)	n	

Above the diagonal, evaluations of the components in *x*-direction were used, significances indicated below the diagonal refer to the components in *y*-direction. Brackets indicate a significance found for *D*_*H*_ but not for SD and vice versa. Kruskal-Wallis, *p ≤ 0.05, **p ≤ 0.01, “n” means not significant.

**Table 2:** Significant differences between balance task results for Higuchi dimensions *D*_*H*_.

	TFOE	TFCE	OFOE	OFCE	TBOE	TBCE	OBOE
**TFOE**		n	*	**	(**)	**	**
**TFCE**	n		(n)	*	(**)	**	**
**OFOE**	**	**		(n)	(**)	**	(**)
**OFCE**	**	**	**		(n)	(**)	*
**TBOE**	*	(n)	(*)	**		*	n
**TBCE**	**	**	(n)	(**)	*		(n)
**OBOE**	**	**	n	*	(**)	n	

Above the diagonal, evaluations of the components in *x*-direction were used, significances indicated below the diagonal refer to the components in *y*-direction. Brackets indicate a significance found for *D*_*H*_ but not for SD and vice versa. Kruskal-Wallis, *p ≤ 0.05, **p ≤ 0.01, “n” means not significant.

**Figure 2:**
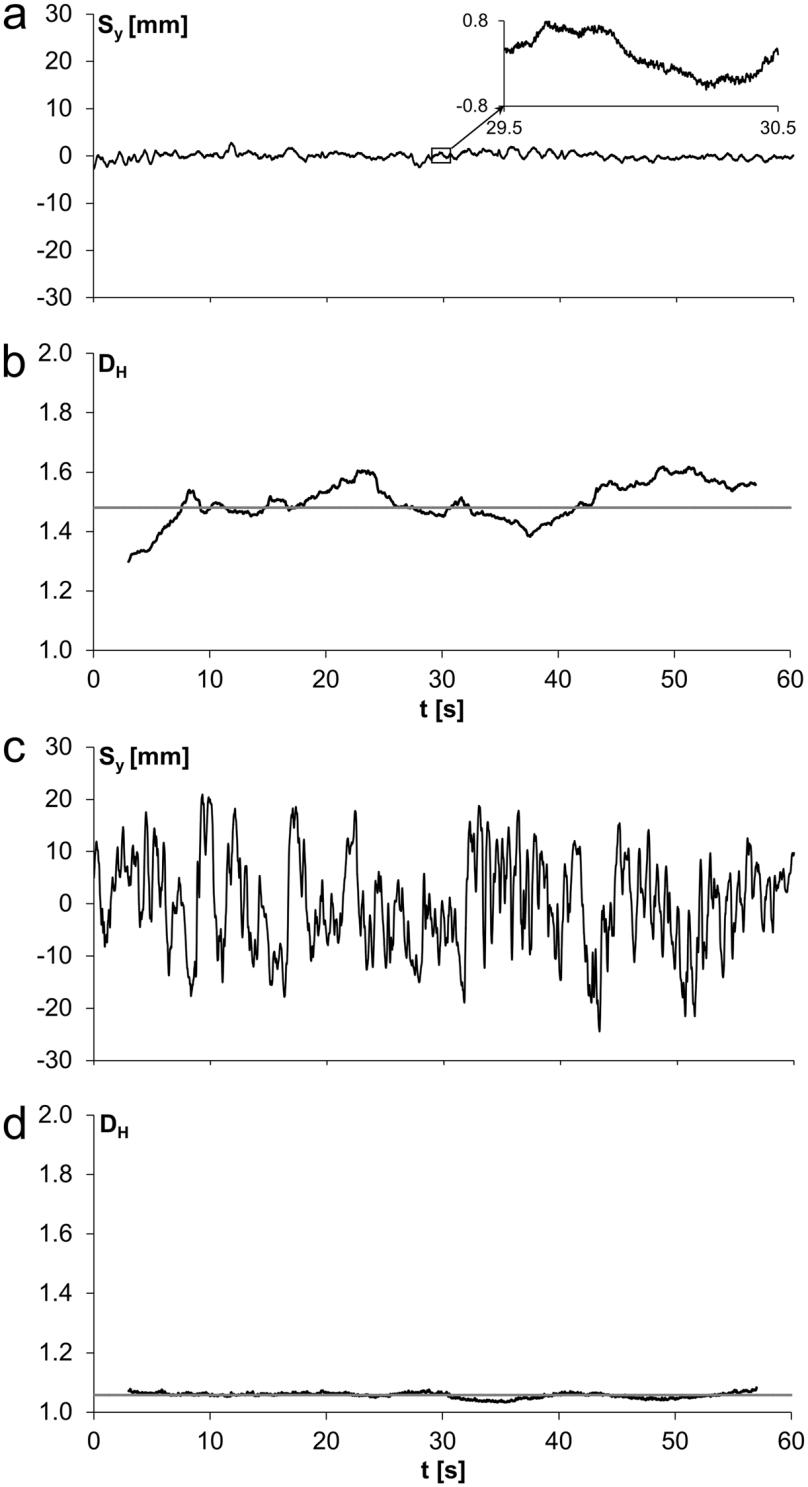
Time signals and Higuchi dimensions. (**a**) Typical COP sway during two-footed stand with open eyes (TFOE) in *y*-direction (*S*_*y*_). 30,000 data points were sampled with a frequency of 500 Hz in 60 s. The insert in the figure shows an enlargement of the signal. This easy task is associated with small elongations. Standard deviation (SD) was 0.74 mm (SD of noise was 0.05 mm) resulting in a signal-to-noise ratio (SNR) of 14.8. (**b**) Higuchi dimension *D*_*H*_ as a function of time (black graph; gliding box length *l* = 3000 data points; *D*_*H*_ ranged from 1.30 to 1.62), and for the entire time signal (grey line; *D*_*H*_ was 1.48). (**c**) Analogous to (**a**), but for the difficult task one-footed stand with closed eyes (OFCE) which is associated with large elongations. SD was 8.82 mm (SD of noise was 0.05 mm) resulting in a SNR of 176.4. (**d**) Analogously to (**b**), but for the signal shown in (**c**). *D*_*H*_ ranged from 1.03 to 1.08 and was 1.06 for the entire time signal.

Figure 2 shows time signals and the according Higuchi dimensions *D*_*H*_ of balance tasks differing in difficulty. The example of one participant is shown. The easy task standing on two feet with open eyes (TFOE) was associated with small elongations in terms of centre of pressure sway in *y*-direction (Fig. 2a), i.e. in medio-lateral direction (and also in *x*-direction, not shown) and with a high Higuchi dimension *D*_*H*_ (Fig. 2b). The black graph shows *D*_*H*_(*t*) using a gliding box length of 3000 data points, corresponding to a time interval of 6 s. The Higuchi dimension *D*_*H*_ for the entire time signal (30,000 data points; 60 s) was 1.48 (grey line) and the values for the gliding Higuchi dimension ranged from 1.30 to 1.62. The elongations measured during standing on one foot with closed eyes (OFCE) of the same participant is shown in Fig. 2c. The standard deviation of the elongations was 11.9 times larger when compared to standing on two feet with open eyes (TFOE). This task is difficult to perform and needs high effort of the participant to remain standing. *D*_*H*_ was very low (close to 1) and almost constant throughout the measurement time (Fig. 2d).

**Figure 3:**
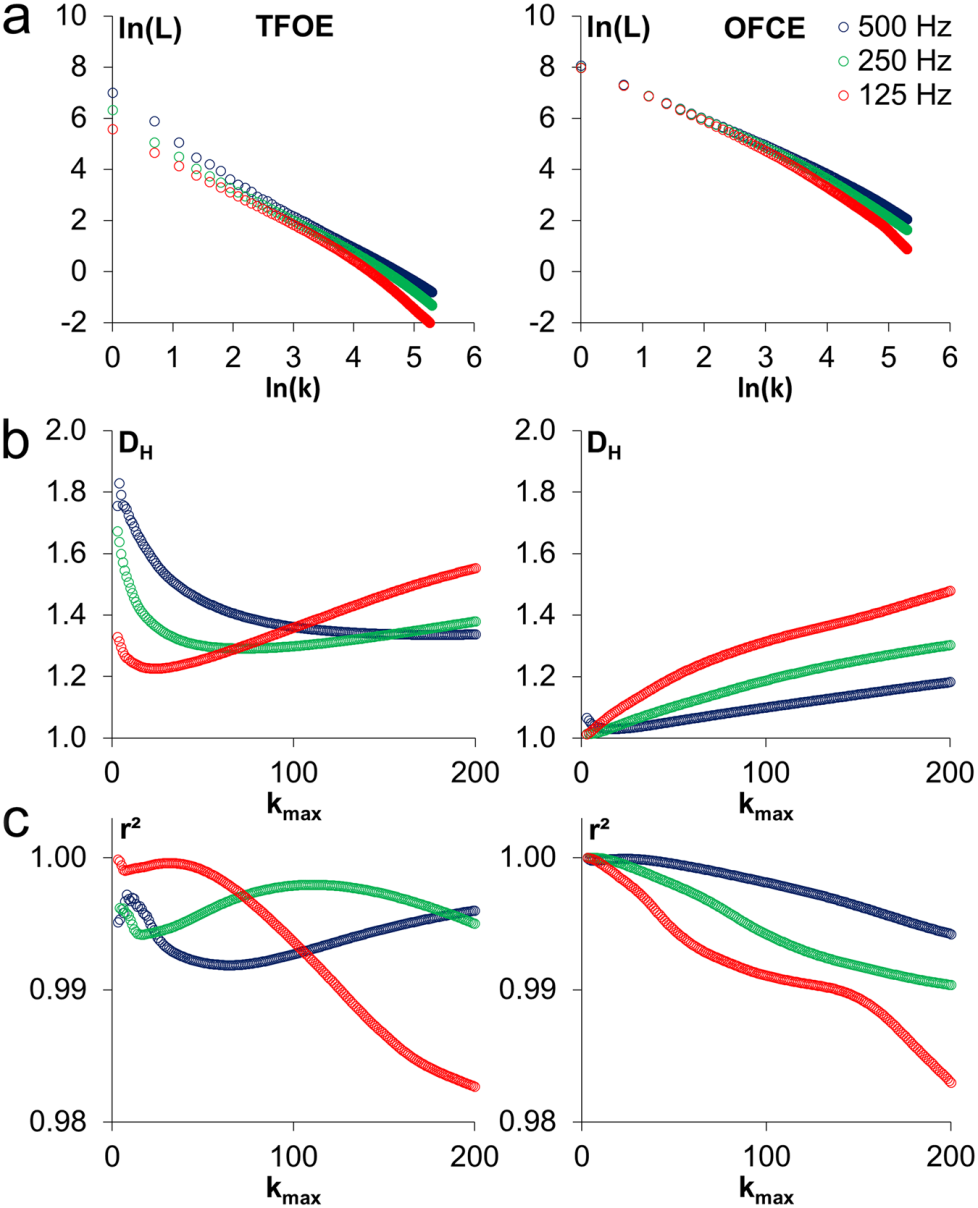
Examples of double logarithmic plots and Higuchi dimensions for *S*_*y*_- time signals of one randomly chosen participant. Left diagrams correspond to the easy task TFOE and right diagrams correspond to the difficult task OFCE. Data was sampled with 500 Hz (blue circles), and results obtained from down-sampled data are shown in green (250 Hz) and red (125 Hz). (**a**) Double logarithmic plots according to equation (6) described in the methods with *k*_*max*_ = 200. (**b**) Higuchi dimensions as a function of *k*_*max*_. (**c**) Coefficients of determination *r* ^2^ according to (**b**). All values are above 0.98.

Double logarithmic plots for the *S*_*y*_-time signals of an easy (TFOE) and a difficult task (OFCE) are shown in Fig. 3a. Sampling frequency was 500 Hz, and results obtained with down-sampled signals are also shown. The Higuchi dimension *D*_*H*_ depends strongly on the choice of the data point interval *k*_*max*_ as can be seen in Fig. 3b. Over the entire range of *k*_*max*_ values, the coefficient of determination *r* ^2^, which is used to determine the linearity in the double logarithmic plot, was larger than 0.98 (Fig. 3c). For comparison, one participant performed the test OFCE in an additional experiment with real sampling frequencies of 500, 250, and 125 Hz. These results (not shown) were highly similar to the results found with down-sampled signals.

**Figure 4:**
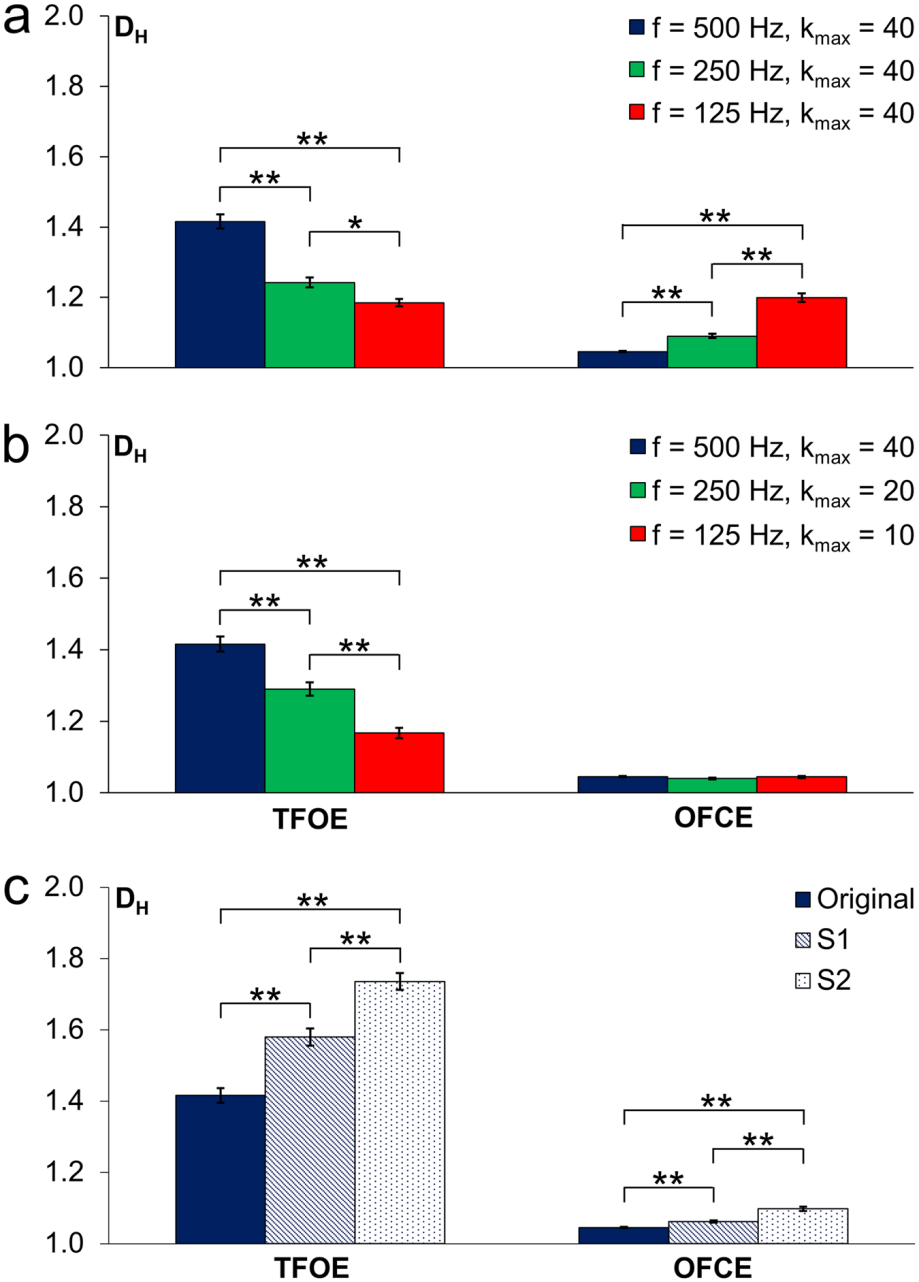
Effect of sampling frequency and noise on the Higuchi dimension for *S*_*y*_- time signals. Left diagrams correspond to the easy task TFOE, and right diagrams correspond to the difficult task OFCE. Mean values and standard errors of means (*n* = 15) of the first attempt of each participant to remain standing over the full 60 s are given. Significant differences (Games-Howell, *p ≤ 0.05, and **p ≤ 0.01) are indicated. (**a**) Effect of sampling frequency on the Higuchi dimension. *D*_*H*_ was computed from time signals taken with a sampling frequency of 500 Hz (blue columns) and *k*_*max*_ was set to 40. *D*_*H*_-values at 250 Hz (green columns) and 125 Hz (red columns) were calculated from down-sampled signals. (**b**) Effects of sampling frequency and data point interval *k*_*max*_. Analogously to (**a**), but *k*_*max*_ was matched to the sampling frequency, such that the effective time interval (0.08 s) remained constant. (**c**) Influence of noise on the Higuchi dimension. The blue columns again correspond to the originally measured signal (500 Hz, *k*_*max*_ = 40), the hatched columns show *D*_*H*_ means when artificial noise (Gaussian random noise, SD = 0.05 mm) was added to each data point of the time signal. Dotted columns show *D*_*H*_ means when the same artificial noise was added again.

For the results shown in Fig. 4, one time signal of each participant performing the tasks TFOE and OFCE was used for computing *D*_*H*_; means and SEMs are plotted. Down-sampling using constant *k*_*max*_ = 40 decreased *D*_*H*_ for the easy task significantly (Fig. 4a), and increased it significantly for the difficult task. The difficult task was associated with a high SD of the sway elongations and a high signal-to-noise ratio (SNR). Matching the sampling frequency with *k*_*max*_ such that the time interval remained constant (0.08 s) resulted in very small (and insignificant) differences of *D*_*H*_ for the difficult task, whereas the reduction of *D*_*H*_ associated with the easy task still remained large and significant (Fig. 4b). Significant differences due to changes in sampling frequency (with and without adaption of *k*_*max*_) are indicated by stars in the column diagrams.

In addition to the choice of the sampling frequency and of *k*_*max*_, the level of noise also influences the value of *D*_*H*_ substantially (Fig. 4c). Artificial (Gaussian random) noise with the same standard deviation as measured experimentally (0.05 mm) was added to the time signals. For the easy task, this additional noise increased the mean *D*_*H*_ (*n* = 15) from 1.42 to 1.58. Addition of noise with twice the standard deviation of the measured noise increased the mean *D*_*H*_ to 1.74. For the difficult task, the addition of noise also increased the mean *D*_*H*_, but differences were much smaller (*D*_*H*_ changed from 1.05 to 1.06 and to 1.10, respectively).

## Discussion

### Human posture control and balancing tasks used to study methodical aspects of nonlinear time signal analyses

Every healthy human can stand on two feet, most of us can stand on one foot and can stand on the balls of both feet without major problems, but trying to stand on one ball for a minute shows that this is quite difficult. Standing on both feet with closed eyes can also be done easily - otherwise we would have problems standing in the dark.

Analysing the balance control is not only of core importance for understanding and quantifying features of the human motor control system, it can also be advantageously applied for analysing the reliability of fractal dimension calculations at various settings of the measurement and of the nonlinear time signal calculation parameters, and further, results from a wide range of task difficulties can be compared. We have developed a series of balance tests for studying the control processes ranging from easily performable tasks like standing on both feet to difficult ones like standing on one ball with open eyes (OBOE), or closed eyes on one foot (OFCE), or on two balls (TBCE). The standard deviations (SDs) of the COP elongations were highest for OFCE, whereas the lowest *D*_*H*_ was found for TB CE in this group of 15 young persons.

Fractal dimension analysis (*D*_*H*_) resulted in 15 significant differences between tasks for the *x*-direction and in 16 for the y-direction, but eight of these 31 differences were not significant when using SD for the analysis. This indicates that the nonlinear time signal analysis is capable of distinguishing between different regulation mechanisms when compared to the analysis of SDs of COP sways.

### Determination of fractal dimensions may fail to assess complexity of a given dynamical process appropriately when effects of noise and parameter settings are not considered adequately

Inadequate choices of nonlinear analysis parameter settings can affect the quantification of the complexity of a process substantially as shown in Figs 3 and 4a,b for the sampling frequency and the choice of *k*_*max*_. The appropriate parameter setting has to be based on both the demands given by the nature of the processes to be studied and by the demands resulting from the mathematical analysis method and its practical application to the given time signals, which can never fulfil the criteria for ideal fractal patterns. Additionally, the SNR always influences the outcome of nonlinear time signal analyses (experimentally measured time signals always contain noise). Noise should be kept as low as possible. This sounds like a trivial statement, but in many publications this important aspect is not sufficiently considered or ignored at all. The SNR must be specified, otherwise comparisons of results obtained by various research groups are not possible in a meaningful way. In cases where the determination of the noise level is difficult, e. g. when studying ECG or EEG signals where the physiologically given signal cannot be switched off, dummy or cadaver measurements should be performed, or, if this is not easily possible, a noise sensitivity analysis should be done by adding artificial noise. For the easy task TFOE, Figure 4c shows that a different fractal dimension (1.58 instead of 1.42, statistically highly significant) already results when artificial noise of the same SD as found in the measured time signal (SD = 0.05 mm) is added. Adding noise with a SD of 0.1 mm resulted in a Higuchi dimension of 1.74 (this is an increase of 76% when considering that the scale for *D*_*H*_ starts out from 1.0). For the difficult task OFCE, addition of noise increased the *D*_H_ value only slightly. These examples show that enormous differences in the results for the fractal dimension can result from small differences of the noise level, which may erroneously be interpreted as a biological effect. We have shown the large impact of Gaussian noise on the Higuchi dimension value as an example. As the fractal dimension computation does not depend on the signal amplitude, other types of noise, even when noise amplitudes are low, can also be expected to influence the outcome substantially, e.g. power line noise, low frequency noise, 1/f noise, or shot noise. Both, the choice of parameter settings for the time signal measurement and also for the nonlinear analysis method applied to it influence the resulting value of the chosen measure of complexity substantially. For all investigations using any nonlinear time signal analysis method to quantify complexity of physiological (or other) processes, we recommend to investigate and protocol the effect of varying noise levels, either by adding artificial noise or/and by changing instrument settings to alter SNR.

### The influence of mismatched parameter settings and increased noise levels are exemplarily shown in this balance analysis using the Higuchi dimension

For the methodical discussion of this nonlinear time signal analysis, we picked out the easy task TFOE and the difficult task OFCE. The crucial measurement parameters are: sampling frequency, SNR, and measurement duration. Sampling frequency of 500 Hz was chosen to include also the fast human biomechanical control processes with reaction times down to 0.04 s in case of trained top level athletes^29^. This would correspond to a maximum frequency of 250 Hz. The sensitivity of nonlinear dynamics analysis of postural control COP measurements on sampling frequency and signal filtering has been shown by Rhea *et al*.^30^. In the study presented here, minimum noise (SD: 0.05 mm) was obtained by setting the force plate measurement range to the lowest possible values for these measurements. The noise signal only contained the electronically determined noise and vibrations transferred via the ground; any additional sources of vibrations (like walking in the lab) were avoided. 60 s was chosen as the standard task duration (30 s was the minimum criterion) to make sure that eventually occurring slower control processes would also be captured.

The crucial parameters for the Higuchi dimension calculation are the number of data points (which should be large) and the choice of the scaling parameter *k*_*max*_. In most attempts, 30,000 data points were captured (minimum criterion were 15,000). This is the maximum that can be applied for this series of balance tasks because a sampling frequency above 500 Hz would not make sense from the physiological perspective, and the test duration had to be limited; otherwise, for the difficult tasks, the number of failed attempts would be too high.

When analysing time signals, *k*_*max*_ represents the maximum temporal data point interval. At 500 Hz sampling frequency, a value of *k*_*max*_ = 40 corresponds to 0.08 s, which is about twice the minimal human reaction time^29^. Up to the *k*_*max*_ value, the linear regressions of the double logarithmic plots (results obtained by one of the 15 test persons for two of the seven tasks are shown in Fig. 3a) should have high coefficients of determination. In all tests performed in this study, at *k*_*max*_ = 40, *r* ^2^ values for all persons and all tests were above 0.984. The point at *k*_*max*_ = 40 is approximately where a visual inspection of the plot, e.g. for OFCE in Fig. 3a, indicates a bending of the line (this visual check is usually used to fix the value of *k*_*max*_). The *D*_*H*_ values shown in Fig. 1c,d were obtained with *k*_*max*_ = 40.

We can try to quantify this approach by using a minimum criterion for *r* ^2^. Figure 3c shows that all *k*_*max*_ values up to 200 result in *r* ^2^ values above a threshold of 0.98. When accepting this threshold of 0.98 for the linearity assessment, all *k*_*max*_ values up to 200 would be acceptable. But, as plotted in Fig. 3b, the *D*_*H*_ value is not independent of *k*_*max*_; therefore, also the choice of a high *r* ^2^-value (e.g. 0.98) cannot determine in an objective way what *k*_*max*_ to use. It just defines the interval within which the *k*_*max*_ value should be chosen.

The final choice of *k*_*max*_ can only be derived from the time course of the biological process studied. In human biomechanical dynamics, the shortest reaction time (minimum of about 0.04 s in highly trained individuals) is a major determinant for mastering a task or for failing. 500 Hz sampling frequency and *k* = 20 (or 125 Hz and *k* = 5) would correspond to a time shift of 20/500 = 0.04 s. For the balance tests presented here, the choice of *k*_*max*_ = 40 covers a *k*-range symmetrically around *k* = 20 which brings the fast balance regulation dynamics into the focus of attention.

For the difficult task OFCE, Figure 4a shows that a reduced sampling frequency without *k*_*max*_ adaption would increase *D*_*H*_ because the (important) fast regulation processes would be missed, whereas *D*_*H*_ remained at the low value when *k*_*max*_ was adapted accordingly (Fig. 4b). For the easy task TFOE (and also for other easy tasks, not shown), we always (with and without *k*_*max*_ adaption) get reduced *D*_*H*_ values when sampling frequency is reduced (Fig. 4a,b) because a lower bandwidth results in a lower noise level, and the loss of high frequency control components is not relevant in easy tasks where rapid reactions do not occur.

## Summary

- Higuchi dimensions resulting from time signal of human control processes (or from other physical processes) are only comparable when the same or similar measurement and *D*_*H*_ calculation parameters are used.
- Changing the sampling frequency can have a significant effect on the *D*_*H*_ (Fig. 4a).
- Changing both, the sampling frequency and using adapted *k*_*max*_ values (such that the effective data point interval remained constant; Fig. 4b) resulted in the same and relevant *D*_*H*_ for the difficult task OFCE where the SNR was high, but it resulted in a significant change of the *D*_*H*_ for the easy task TFOE, where the SNR was low.
- The choice of *k*_*max*_ has a significant impact on the *D*_*H*_ value. A change of the *k*_*max*_ value may even invert the order of *D*_*H*_ values, particularly when SNR of the time signal is low (Fig. 3b). It is of paramount importance to choose the effective data point interval in accordance with the characteristic dynamics of the process.
- *D*_*H*_-computations are very sensitive to the SNR (Fig. 4c).
- Without analysing the sensitivity to noise in a given setting, it cannot be distinguished whether a high *D*_*H*_ is due to a more pronounced stochastic component of the studied process or because of a low SNR.
- With increasing noise, *D*_*H*_ differences between easy and difficult (complex) control processes increase. Our experiments have shown that adding the same noise level increases the *D*_*H*_ of easy tasks (where SNR is low) in a more pronounced way when compared to difficult tasks (Fig. 4c).

With the set of parameters used in this study, the standard errors of the means for *D*_*H*_ (n = 15, i.e. 15 test persons performed each test) were remarkably small in all seven tests (Fig. 1c,d).

Nonlinear time signal analysis was capable of distinguishing between different regulation determining mechanisms than the analysis of SDs of COP sways. This may enable more differentiated diagnoses of balancing problems associated with diseases on the one side, and also an improved analysis of high balance performance levels of trained persons on the other.

When patients suffering from dizziness or other posture control deficiencies, or when trained balance acrobats performed the test series, pronounced differences from the means shown in Fig. 1c,d can be expected. The test series and the measurement and data evaluation procedure presented here for the first time can be used as a comprehensive approach to study balancing abilities and intervention effects in persons ranging from top level athletes to patients suffering from dizziness (in the latter case, a subset of the balance tasks will be sufficient). Usage of the balance control test series described here as a standardised approach could lead to a much better comparability of balance variable measurements, which is currently lacking^10^.

Fractal dimension analyses can detect signal features that cannot be derived from linear time signal analyses, but a standardization of the measurement and the fractal dimension computation parameter setting is necessary because results obtained with different parameter settings may easily lead to incoherent results, and even trends obtained with one set of parameters can turn upside-down with other settings. This can also happen because of changing noise levels that may be present when different experiments (tasks) are compared. Results that have been published so far without specification of the measurement and dimension calculation parameter settings, without specification of the SNR in the time signals, and without a noise sensitivity analysis are not comparable to results obtained by others - and therefore without scientific value.

## Methods

### Study design

All 15 participants performed the balance task series consisting of seven balance tasks. Each participant performed the series three times, with pauses of 90 s between each series. The order of the balance tasks was always the same: standing on two feet flat on the ground with open eyes (TFOE), two feet flat on the ground with closed eyes (TFCE), one foot flat on the ground with open eyes (OFOE), one-foot flat on the ground with closed eyes (OFCE), two balls of the feet with open eyes (TBOE), two balls of the feet with closed eyes (TBCE), and one ball of the foot with open eyes (OBOE). Test duration was 60 s, but attempts interrupted after at least 30 s were also accepted for statistical analyses. For the analysis of parameter settings and noise effects, the first attempt was used or, in case that there was no attempt lasting 60 s the test with the longest duration was taken. Within a series, only after OFCE and after the three tasks on the ball(s) pauses of 30 s were inserted. All participants were instructed to stand upright on the force plate without shoes as stable as possible and without talking. For the tasks with both legs, feet had to be positioned at the width of the pelvis. For the tasks on one leg, they were allowed to choose a preferred leg, but they had to remain with this leg.

### Higuchi dimension

The Higuchi dimension is a fractal dimension characterizing the complexity of time signals directly in the time domain^28^. The values of the Higuchi dimension for one dimensional time signals fall into the closed interval [1, 2]. Periodic signals, e.g. sinusoidal signals, have a dimension *D*_*H*_ = 1, whereas purely random signals (noise), have a dimension *D*_*H*_ = 2.

A discrete time signal, e.g. a digitized analogue signal, consists of a finite set of data points:

1 $$S:x(1),\,x(2),\,x(3),\ldots ,x(N),$$

with *N* being the total number of data points. From the given discrete time signal *S*, new time signals *S*(*m*, *k*) are constructed, with *m* indicating the initial data point and *k* the data point interval.

2 $$S(m,k):x(m),x(m+k),x(m+2k),\ldots ,x(m+\lfloor \frac{N-m}{k}\rfloor k)(m=1,2,\ldots ,k),$$

where $\lfloor \,\rfloor$ denotes the floor function which rounds the number down to the nearest integer. For each *S*(*m*, *k*), the normalized lengths *L*_*m*_(*k*) are calculated as follows:

3 $${L}_{m}(k)=\frac{1}{k}\{(\sum _{i=1}^{\lfloor \frac{N-m}{k}\rfloor }|x(m+ik)-x(m+(i-1)k)|)\frac{N-1}{\lfloor \frac{N-m}{k}\rfloor k}\},$$

where *m* and *k* are integers and $\frac{N-1}{\lfloor \frac{N-m}{k}\rfloor k}$ is a normalization factor. For each *k* the mean length *L*(*k*) is calculated

4 $$L(k)=\frac{1}{k}\sum _{m=1}^{k}{L}_{m}(k).$$

If the power law

5 $$L(k)\propto {k}^{-{D}_{H}}$$

is fulfilled, the given time signal *S* is fractal with the dimension *D*_*H*_. If *L*(*k*) is plotted against *k* on a double logarithmic scale, the data should fall on a straight line with the negative slope

6 $${D}_{H}=-\frac{{\rm{d}}\,\mathrm{ln}(L(k))}{{\rm{d}}\,\mathrm{ln}(k)}$$

defined as the Higuchi dimension *D*_*H*_. For measured data, linear regressions with coefficients of determinations *r* ^2^ close to 1 are required to classify the time signal to be fractal. However, *D*_*H*_ depends on *k*_*max*_ and therefore, it is important to apply a criterion for choosing *k*_*max*_. The slope should be as linear as possible for all investigated time signal. For this study, *k*_*max*_ = 40 was chosen; all coefficients of determination *r* ^2^ were above 0.984. Sampling frequency was 500 Hz. Down-sampled time signal were also investigated where *k*_*max*_ was set to 20 at 250 Hz and *k*_*max*_ was set to 10 at 125 Hz. The Higuchi dimension *D*_*H*_was computed for the entire time signal as well as for intervals *I*(*n*, *n* + *l*) with *n* = 1,2, …, *N* − *l* throughout the entire time signals yielding a function of time of the Higuchi dimension (“gliding *D*_*H*_”). The box length *l* was set to 3000 data points. Higuchi dimensions were computed with the open access software IQM^31^.

### Measurement system

Ground reaction forces were measured with a piezoelectric force measurement system (Kistler Instrument Corporation, Switzerland, platform: 9286AA, data acquisition: 5691A). Measurement ranges were set to the minimum necessary to detect the force amplitudes occurring during the experiments (*F*_*x*_: 130N; *F*_*y*_: 130N; *F*_*z*_: 568N; *F*_*z*_ was found to be linear up to 1000N) to keep the electronically determined noise level as low as possible. Resolution was 16 bit. Data was recorded with a sampling frequency of 500 Hz over 60 s. This corresponds to 30,000 force vectors at the four measurement sites of the force plate. Kistler BioWare software computes the time signals of the resulting ground reaction forces, the moments, and the coordinates of the centre-of-pressure (COP) in the measurement plane. Kister Bioware provides post-processing filters, but they were not applied. Post-processing filters were only used for the analysis of down sampled signals (two-point moving average).

During the balance tests, the participants looked into the *x*-direction. COP sway time signals *S*_*x*_(*t*) and *S*_*y*_(*t*) were used for mapping the anterior-posterior (*S*_*x*_) and medio-lateral (*S*_*y*_) movement of the body. In contrast to the vertical ground reaction force *F*_*z*_, the COP elongations in *x*- and *y*-direction were not sensitive to heart beats.

Noise, drift, and cross-talk of the measurement system were analysed for *S*_x_(*t*) and *S*_*y*_(*t*) signals by using calibrated weights on the force plate. Ten measurements over 60 s were performed. The standard deviation of each of the ten noise measurements was used to represent the noise level; maximum was 0.03 mm for *S*_*x*_ and 0.05 mm for *S*_*y*_. *D*_*H*_ of the noise signals were close to 2.0 [1.95, 2.00]. Drifts ranged from −0.06 mm to 0.11 mm, but did not influence the value of the Higuchi dimension *D*_*H*_ (this was tested experimentally by adding an artificial drift). Cross talk effects of *F*_*z*_ on *F*_*x*_ and *F*_*y*_ were less than 0.12%. The effect on COP shifts was smaller than 0.06 mm, i.e. negligible at the maximal measured force *F*_*z*_ = 900N. This small cross-talk did not alter the *D*_*H*_values.

### Down-sampling

The analysis of sampling frequencies on the outcome could be done by setting the sampling frequency of the measurement system. This would necessitate three measurements of each task to compare three sampling frequencies. However, this would sum up to very long measurement sessions, and the limited performance reproducibility of the test persons (including fatigue or possible training effects) would complicate the interpretation of the results. Therefore, down-sampled time signals with effective sampling frequencies of 250 Hz and 125 Hz were constructed by applying two-point moving average filtering.

### Simulated noise

Artificial randomly distributed noise was added to experimentally gained time signals. In order to ensure comparability, the standard deviations of the noise values were set to 0.05 mm and 0.10 mm (0.05 mm was the average standard deviation of the measured noise signals). The artificial noise simulations were obtained by adding a random value to each data value, but not by adding a separate noise signal which might have contained higher frequency components.

### Participants

Six females and nine males participated in the study. Means and standard deviations of the participants were: age (25 ± 4) y, body height (176 ± 8) cm, body mass (65.9 ± 8.6) kg. Ethical approval was given by the Ethics commission of the Medical University of Graz (20-295ex 08/09). All methods were performed in accordance with the relevant guidelines and regulations. Informed consent was obtained from all participants.

### Statistics

Anthropometric data of participants are given in means ± SD. Normal distribution was fulfilled according to the Shapiro-Wilk test (*p* ≤ 0.05). In column diagrams, mean values and standard errors of means (SEM) are shown.

Not all balance data sets (Fig. 1, Tables 1 and 2) were normally distributed (Shapiro-Wilk test, *p* ≤ 0.05) and therefore the Kruskal-Wallis test, including post-hoc tests, was applied to determine significances (*p ≤ 0.05, **p ≤ 0.01) between the cases.

The two balance data sets in Fig. 4, the down-sampled data, the k-value adapted data, and the data sets with added noise were normally distributed (Shapiro-Wilk test, *p* ≤ 0.05). Welch’s-ANOVA was applied. Variance homogeneity was not fulfilled (Levene’s test, *p ≤ 0.05). Games-Howell post-hoc tests were used to determine significances (*p ≤ 0.05, **p ≤ 0.01) between the cases. Software SPSS (Version 23) was used.
